# Antioxidant Properties of Chokeberry Products—Assessment of the Composition of Juices and Fibers

**DOI:** 10.3390/foods12214029

**Published:** 2023-11-05

**Authors:** Ewa Olechno, Anna Puścion-Jakubik, Jolanta Soroczyńska, Katarzyna Socha, Monika Cyuńczyk, Małgorzata Elżbieta Zujko

**Affiliations:** 1 Department of Food Biotechnology, Faculty of Health Science, Medical University of Białystok, Szpitalna 37 Street, 15-295 Białystok, Poland; ewa.olechno@sd.umb.edu.pl (E.O.); monika.cyunczyk@umb.edu.pl (M.C.); 2 Department of Bromatology, Faculty of Pharmacy with the Division of Laboratory Medicine, Medical University of Białystok, Mickiewicza 2D Street, 15-222 Białystok, Poland; jolanta.soroczynska@umb.edu.pl (J.S.); katarzyna.socha@umb.edu.pl (K.S.)

**Keywords:** chokeberry, juices, fibers, conventional products, organic products, minerals, polyphenols, flavonoids

## Abstract

Chokeberry fruits are a rich source of bioactive ingredients and their beneficial effect on the body has been proven in the literature. They contain antioxidants such as polyphenols (anthocyanins, procyanidins, phenolic acids, flavonols and flavanols) but also other essential substances with health-promoting potential, such as vitamin C and elements. Providing the right amount of these ingredients is very important for maintaining health and preventing the effects of oxidative stress. The aim of the study was to assess the content of antioxidant elements (magnesium—Mg) and trace elements (copper—Cu, iron—Fe, manganese—Mn, selenium—Se and zinc—Zn), with the antioxidant potential being measured using the FRAP method, along with total anthocyanin, total flavonoid and total polyphenol content (TPC) in 25 chokeberry juices and 6 chokeberry fibers sourced from conventional and organic farming. All chokeberry juices and chokeberry fibers available on the Polish market at that time were ordered for testing. The studied juices came from concentrate (FC) and not from concentrate (NFC). Taking into account the mineral content, it was shown that both chokeberry juices and fibers contained the highest amount of Mg and the lowest amount of Se. The FRAP value was significantly higher (*p* < 0.05) in organic juices compared to conventional ones as well as being higher (*p* < 0.05) in NFC juices compared to FC juices. NFC juices were also characterized by their higher concentrations of TPC, total flavonoid and total anthocyanin levels (*p* < 0.05) compared to FC juices. Consumption of 100 g of chokeberry juice can cover from 149.5 to 3177.0% of the daily requirement for Cu, 6.8–32.4% for Mn, 2.8–6.1% for Mg, 0.9–7.4% for Se, 0.2–3.7% for Fe, 0.3–1.2% for Zn and 8.3–34.5% for vitamin C. In turn, the consumption of 10 g of fiber can cover 4.3–32.0% of the daily requirement for Fe, 0.6–9.0% for Se, 3.7–8.2% for Cu, 2.2–3.8% for Mg, 0.6–9.0% for Se, 0.9–8.5% for Zn and 0.5–0.7%% for vitamin C. Chokeberry products can be a valuable component of a healthy diet.

## 1. Introduction

Chokeberry fruits are known for their health-promoting properties. Their lipid-lowering, hypotensive and hypoglycemic effects have been confirmed in meta-analyses [1,2]. Their possibilities in the treatment and prevention of diabetes, liver diseases, cardiovascular diseases and neurodegenerative diseases are emphasized [3]. Chokeberry fruits owe their properties to their antioxidant content. They contain antioxidants, in particular, polyphenols (anthocyanins, procyanidins, phenolic acids, flavonols and flavanols) [4]. However, it is worth emphasizing, that chokeberry fruits also contain other substances with antioxidant and anti-inflammatory potential, such as vitamin C, magnesium (Mg), copper (Cu), iron (Fe), manganese (Mn), selenium (Se) and zinc (Zn) [4,5,6]. It is well known that antioxidants are extremely important in prevention the effects of oxidative stress [7]. Oxidative stress is associated with excessive production of reactive oxygen species and contributes to cellular damage in our bodies [8]. The formation of reactive oxygen species is a natural process in the human body. Our organism has internal mechanisms, including antioxidant enzymes, that help maintain the antioxidant–oxidant balance [7]. However, nowadays, this balance can be disturbed as a result of various factors such as a highly processed diet, exposure to environmental pollution, alcohol consumption or smoking. Therefore, it is extremely important to consume products rich in antioxidants, such as chokeberry products, which could prevent the effects of oxidative stress [9]. Many different products are produced from chokeberry fruits, among others, juices, jams, syrups, teas, supplements and fibers [4]. Due to the fact that chokeberry fruits can be eaten in different forms, it could differ in its biochemical composition, including the antioxidant content. The amount of these ingredients may also be influenced by the variety and degree of ripeness, cultivation method, soil and climatic conditions, as well as technological processes used during production, such as heating or drying [10,11,12,13].

The aim of the study was to assess the content of health-promoting compounds in chokeberry juices and fibers available on the Polish market. We assessed the antioxidant potential, total polyphenol content (TPC), total flavonoids, total anthocyanins, vitamin C and antioxidant elements Cu, Fe, Mg, Mn, Se and Zn. The great advantage and novelty of our research is the fact that the scope of the products tested included all juices and fibers available on the Polish market. To our knowledge, these products have not been studied so extensively so far.

## 2. Materials and Methods

### 2.1. Materials

The research material consisted of 25 chokeberry juices (100%) without additives. All juices were pasteurized. A total of 15 juices were organic juices, while 10 juices were conventional juices. Among these juices, 20 juices were not from fruit concentrate (NFC), and 5 juices were from fruit concentrate (FC). Additionally, six chokeberry fibers available on the market were also tested—three organic and three conventional. The products were purchased at the Polish local market and in Polish online stores. All chokeberry juices and chokeberry fibers available on the Polish market at that time were ordered for study. All samples came from 3 different lots.

### 2.2. Methods

The contents with antioxidant potential, total content of polyphenols (TPC), total flavonoids, total anthocyanins, vitamin C and elements Cu, Fe, Mg, Mn, Se and Zn were determined in chokeberry juices and fibers. Analyses of the tested substances were performed in three repetitions.

In order to determine antioxidant properties, chokeberry juices were diluted with ultrapure water and chokeberry fibers were extracted with methanol/acetone mixture as described in a previous publication [14]. The obtained extracts were used to determine FRAP, polyphenol, total flavonoid and total anthocyanin content. Measurements were performed on a UV spectrophotometer (Shimadzu, Kyoto, Japan).

The minerals content was determined using the atomic absorption spectrometry (AAS) method, after previous microwave mineralization [15]. The data shown is the average of two measurements.

#### 2.2.1. Total Anthocyanins

Total anthocyanin content was determined by the Giusti and Wrolstad method [16]. This method is based on differences in absorbance (520 nm and 700 nm) of samples with potassium chloride buffer (pH = 1.0) and samples with sodium acetate buffer (pH = 4.5) after 15 min of incubation at room temperature. The total anthocyanin content was expressed as cyanidyn-3-glucoside (mg Cy-3-GL/kg).

#### 2.2.2. Total Flavonoids

The total flavonoid content was based on the formation of aluminum-flavonoid complexes [17]. Briefly, 2% AlCl_3_ in methanol was mixed with the same volume of the sample. The absorbance was measured at 415 nm after 10 min of incubation at room temperature against a blank consisting of a sample with methanol without AlCl_3_. The total flavonoid content was expressed as quercetin equivalents (mg QE/kg).

The purpose of determining the content of total flavonoids and total anthocyanin content was to present in more detail what part of polyphenols the above-mentioned compounds may constitute.

#### 2.2.3. Polyphenols

The total polyphenol content was determined using the Folin–Ciocalteau reagent [18,19]. The yellow reagent is oxidized using phenolic compounds occurring in samples in the presence of sodium carbonate and creates blue complexes after reduction. The absorbance was measured at 765 nm after 30 min incubation at room temperature against a blank without sample. The total polyphenol content was calculated from the standard curve and expressed as gallic acid equivalents (mg GAE/kg).

#### 2.2.4. Ferric Ion Reducing Antioxidant Potential (FRAP)

The ferric ion reducing antioxidant potential (FRAP) was determined according to Benzie and Strain method [20]. This method is based on the reduction in the Fe^3+^ ions in the form of a complex with 2,4,6-Tri(2-pyridyl)-s-triazine (TPTZ)—TPTZ-Fe^3+^ to the Fe^2+^ ions—TPTZ-Fe^2+^ in the presence of antioxidants in sample. The absorbance (an intense blue color) was measured at 593 nm after 4 min incubation at 37 °C against a blank without sample. The FRAP was calculated from the standard curve and expressed as mmol/kg.

#### 2.2.5. Determinations of Minerals Content

In order to determine the mineral content, microwave mineralization of samples was carried out in a closed system. The samples were weighed to the nearest 1 mg and spectrally pure concentrated nitric acid 69% (4.0 mL, 69% HNO_3_, Tracepur, Merck, Darmstadt, Germany) was added. The mineralization process was carried out in a closed-loop microwave system (Speedwave, Berghof, Eningen, Germany). The samples were transferred to vessels using ultrapure water prepared using a Simplicity 185 device (Millipore, Burlington, VT, USA). The exact sample volumes after mineralization were recorded.

The content of selected elements in the samples was determined using AAS method using the Z-2000 apparatus (Hitachi, Tokyo, Japan). Depending on the ranges of the standard curves, the mineralizates were appropriately diluted with ultrapure water. To determine the content of Cu, Mn and Se, the flameless AAS technique with electrothermal atomization in a graphite cuvette was used—during the determination of the Se content, a palladium–magnesium matrix modifier (Merck, Darmstadt, Germany) was added (Pd concentration: 1500 mg/L and Mg concentration: 900 mg/L), and for the measurement of Mn, magnesium nitrate (Mg(NO_3_)_2_ concentration: 100 mg/L, Sigma-Aldrich, Merck, Darmstadt, Germany) as a modifier was used. The AAS flame technique in an acetylene–air flame with Zeeman background correction was used to determine the content of Fe, Mg and Zn. In the case of Mg content determination, a masking agent was used—1% lanthanum chloride (LaCl_3_, Sigma-Aldrich, Merck, Darmstadt, Germany). To validate the method, certified reference material was used: Tea Leaves—INCT–TL–1—Polish certified reference material for multielement trace analysis (Institute of Nuclear Chemistry and Technology, Warsaw, Poland) [15].

#### 2.2.6. Vitamin C

The sum of L-ascorbic acid and dehydroascorbic acid was determined using the high-performance liquid chromatography (HPLC) method with UV detection, wavelength: 254 nm (Perkin Elmer, Waltham, United States). Dehydroascorbic acid is reduced to L-ascorbic acid using dithiothreitol (DTT). The results were expressed in mg/100 g of product [21].

#### 2.2.7. Comparison to Reference Intake Values

Chokeberry juices and fibers can be consumed on their own, but they also have the potential to be used, among other things, for the production of dietary supplements and functional foods. Therefore, we compared the obtained results to the reference intake values (RIV) per day, which are: vitamin C: 80 mg, Mg: 375 mg, Fe: 14 mg, Zn: 10 mg, Cu: 1 mg, Mn: 2 mg and Se: 55 µg [22].

#### 2.2.8. Statistical Analysis

Statistical analyses were created using the Statistica v.13.3 software (StatSoft, Krakow, Poland). Kruskall–Wallis Analysis of Variance (ANOVA) with post hoc analysis to compare the content of the studied components between the subgroups of products was performed. The assessment of statistical differences was carried out using the Mann–Whitney U test (*p*-values < 0.05 denote statistical significance).

## 3. Results and Discussion

The results obtained are presented in Table 1, Table 2, Table 3, Table 4 and Table 5. Table 1 shows the mineral content of the studied chokeberry juices. Table 2 presents antioxidant properties of the studied chokeberry juices—total anthocyanins, total flavonoids, FRAP, TPC and vitamin C content. Table 3 shows the mineral content in chokeberry fibers. Table 4 includes the antioxidant content in the studied chokeberry fibers. Table 5 presents the percentage of RIV of the studied elements (Cu, Fe, Mg, Mn, Se and Zn) for a portion of juice or fiber. Table S1 shows the results of the studied components in chokeberry products and other plant products obtained by other authors.

All studied chokeberry juices contained the highest amounts of Mg (152.84 mg/kg) and Cu (65.64 mg/kg), then Mn (3.81 mg/kg), Fe (1.04 mg/kg), Zn (0.59 mg/kg) and Se (15.78 µg/kg). In both conventional and organic juices, the content of individual elements was at a similar level and no statistically significant differences were found. The FRAP values in all juices ranged from 41.79 to 85.66 mmol/kg in juices from traditional cultivation and from 56.14 to 97.41 mmol/kg in the case of juices from organic cultivation. This value was significantly higher (*p* < 0.05) in organic juices compared to conventional ones (65.25 and 75.56 mg/kg). The TPC level was similar in traditional and organic juices, 3285.00–4633.00 mg GAE/kg for traditional juice and 3375.00–4566.00 mg GAE/kg for organic juice, respectively. The cultivation method also had no impact on the content of total flavonoids and total anthocyanins. The level of total flavonoids in conventional and organic juices did not differ: 507.90–726.90 mg QE/kg for conventional juice and 511.50–947.90 mg/kg for organic juice (*p* > 0.05), respectively. The total anthocyanin content for all studied juices ranged from 156.70 to 297.20 mg Cy-3-O-GL/kg of juice. No differences were found depending on the cultivation method.

Some differences were found between NFC and FC juices. The FRAP value was higher (*p* < 0.05) in NFC juices compared to FC juices (75.120 and 56.14 mg/kg). NFC juices were also characterized by higher concentrations of TPC, total flavonoids and total anthocyanins levels (*p* < 0.05) than those found in FC juices. In the case of vitamin C, no statistically significant differences were observed.

No significant differences were found between chokeberry fibers from organic and conventional cultivation. In the case of minerals, all studied fibers contained the highest amount of Mg (1014.65 mg/kg), then Fe (283.84 mg/kg), Mn (36.13 mg/kg), Zn (17.13 mg/kg), Cu (6.17 mg/kg) and Se (61.03 µg/kg). There were no significant differences (*p* > 0.05) in the content of FRAP, polyphenols, total flavonoids, total anthocyanins and vitamin C in organic fibers compared to conventional fibers.

Table 5 shows that the tested chokeberry products cover the requirement for Cu, Mg, Mn, Se, Zn and vitamin C. We have adopted the recommended portion sizes: 100 g for chokeberry juice and 10 g for chokeberry fiber. These amounts are consistent with the daily recommendations of the manufacturers of these products. When determining the coverage of daily intake, we relied on the daily RIV for vitamins and minerals [22]. Taking into account the coverage of the requirement for individual minerals by a portion of the product, we can note that chokeberry products can be a good source of Cu and Fe in the diet. Consumption of 100 g of chokeberry juice will cover as much as from 149.5 to 3177.0% of the daily Cu requirement. In turn, a portion of fiber will provide from 4.3 to 32.0% of the daily Fe requirement. In the case of other elements, a serving of juice will cover 6.8–32.4% of the daily requirement for Mn, 2.8–6.1% for Mg, 0–7.4% for Se and 0.2–3.7% for Fe. In turn, the consumption of 10 g of fiber can cover 15.8–20.3% of the daily reference intake for Mn, 3.7–8.2% for Cu, 2.2–3.8% for Mg and 0.6–9.0% for Se. The studied chokeberry products provide Zn in the smallest amounts—0.3–1.2%% for the juice portion and 0.9–8.5%% for the fiber portion. Chokeberry products may also provide certain amounts of vitamin C. Juices will cover 8.3–34.5%% of the daily necessary dose of vitamin C and fibers 0.5–0.7%% of daily intake.

In the study by Pavlovic et al. (2015), it was noticed that chokeberry juices also contained the most Mg, followed by Fe, Mn, Zn, Cu and Se [23]. In the study by Cindric et al. (2017), other macroelements and microelements were also examined, but Mg was the element found in the highest amount in chokeberry juices [24].

The median content of Mg in the studied chokeberry juices was 152.84 mg/kg. Mg is a macroelement that is a cofactor of over 300 enzyme systems. It plays a role in regulating blood pressure, affects neuromuscular conduction, DNA synthesis, proper functioning of mitochondria and insulin metabolism [25]. Deficiency of this macroelement may contribute to increased oxidative stress, among other things, by increasing the production of reactive oxygen species in mitochondria and reducing antioxidant defense systems [26]. It is associated with increased inflammation in the body and higher risk of many diseases and metabolic disorders [26,27]. The median of Mg content in all chokeberry juices in our study was lower than in chokeberry juices in the study by Torovic et al. (2023) (about 479 mg of Mg/kg juice, respectively) and Pavlovic et al. (2015) (about 251 and 589 mg of Mg/kg juice, respectively) [23,28]. Comparing the content of the studied elements in chokeberry juices in our study to other fruit juices, the median Mg content was lower than in bilberry juice [28] and higher than in apple, kiwi, pear, cherry [29], peach, apricot and orange juices [29,30]. Pineapple juice contained a similar Mg content compared to chokeberry juices in our study—about 140.42 mg/kg [29]. In our study, chokeberry fibers had a much higher Mg content than juices. The median was 1014.648 mg/kg of fiber, which results from the form of this product and the concentration of ingredients. It is worth emphasizing that chokeberry products differ in their mineral content. For example, fresh chokeberry fruits could contain a higher mineral content than juices and infusions [23,24,28]. We could not compare the studied chokeberry fibers to other products of this type due to the fact that, to our knowledge, there is a lack of research on this type of product.

Mn is a trace element, which is an activator and a component of enzymes such as superoxide dismutase in mitochondria, alkaline phosphatase, pyruvate carboxylase, glucosyltransferase and glutamine synthetase. Mn is found in tea, nuts, cereal products, legumes and seafood, and in small amounts in some fruits and vegetables [31]. The median Mn content in the studied chokeberry juices was 3.813 mg/kg and it was similar or lower compared to the chokeberry juices in other studies. In the study by Torovic et al. (2023), it was 3.1 mg/kg and 2.98–11.77 mg/kg in the study by Pavlovic et al. (2015) [23,28]. Chokeberry juices in our study also had a lower Mn content compared to bilberry juice in the study by Torovic et al. (2023) (about 0.53 mg/kg) and juices in the study by Harmankaya et al., 2011 (from 0.100 mg/kg for orange juice to 0.530 mg/kg for cherry juice) [30]. Juices in the study by Dehelean et al. (2013) also contained lower Mn content—from 0.0596 mg/L in the case of peach juice to 0.343 mg/L for apple juice [29]. The median of Mn content was higher in chokeberry fibers (36.13 mg/kg, respectively) than in chokeberry juices.

Fe is a trace element that takes part in the transport of oxygen in hemoglobin and myoglobin. It is also responsible for energy metabolism in cells and electron transfer in mitochondria [32]. Iron can be an antioxidant and prevent the formation of reactive oxygen species if it is delivered in suitable amounts. This element is part of catalase—an enzyme involved in oxidative reactions. Fe occurs in two oxidation states: in the form of Fe^2+^ and Fe^3+^ ions, therefore, it can be both an electron donor and acceptor [32,33]. It is found in products in the heme form (in animal products) and in the non-heme form (in plant products). Heme Fe is characterized by better bioavailability and is found in the largest amounts in red meat, offal and egg yolks. Non-heme iron has low bioavailability. It is found in plant products such as legumes, cereal products, nuts and seeds, as well as in some dark green leafy vegetables such as parsley and spinach [32]. The median of Fe in studied chokeberry juices was 1.039 mg/kg (0.307–5.236 mg/kg of juice). In Torovic et al.’s (2023) study, Fe levels in chokeberry juices were similar to those obtained in our study, respectively, 75 mg/kg of juice. In turn, in Pavlovic et al.’s study (2015), they obtained higher Fe values, 7.2–25.2 mg/kg, respectively [23,28]. If we compared our studied juices to other fruit juices, bilberry, peach, apricot and cherry juices had higher iron content [28,30]. In turn, orange juices had both lower and higher Fe content (0.82–1.4 mg/kg) [30]. The Fe content of fiber was 283.84 mg/kg and it is higher than in chokeberry juices. However, it should be emphasized that in plant products there is non-heme iron, which it is less absorbed [34]. Therefore, it seems that the products we tested will not be a good source of iron.

Cu is a part of ceruloplasmin in the body, which catalyzes the oxidation of Fe. It is also a cofactor for some antioxidant enzymes, including superoxide dismutase, tyrosinase and oxidases. The sources of Cu in the diet will be legumes, cereal products, nuts and seeds [35]. The median of Cu in studied chokeberry juices was 65.64 mg/kg (14.95–317.70 mg/kg). Torovic et al. (2023) obtained lower Cu content in chokeberry juices, respectively, 0.06 mg/kg [28]. In turn, in the study by Pavlovic et al. (2015), the authors also observed lower Cu content: 0.68–4.51 mg/kg [23]. The previously mentioned fruit juices had lower Cu content in other studies [29,30]. The Cu content in studied fibers was 6.173 mg/kg and it is lower than chokeberry juices in our study, but higher than the Cu content in other chokeberry products [28]. Due to the fact that the amount of copper that the chokeberry products we tested can provide is high, it should be mentioned that the bioavailability of copper from the diet in inhabitants of industrialized countries ranges between 30 and 40% [36]. The absorption of copper depends on many factors, such as the form in which copper occurs, the content of other minerals in the diet, organic acids and the presence of fiber and protein in the diet [34,37]. The body has mechanisms that regulate copper absorption. As a result of a large intake of copper at one time, the absorption of this element in the digestive tract decreases and it is excreted. Moreover, copper from food comes in an organic form, which is processed by the body differently than copper in its inorganic form. Copper is also more bioavailable when sourced from animal products than from plant products [34]. It seems that copper toxicity primarily occurs when people consume excess copper from drinking water or dietary supplements, where it occurs in an inorganic form [34,37]. Nevertheless, the products we tested are a rich source of copper, and caution should be exercised, especially in the case of older people whose metal metabolism may be altered. Moreover, the element content is strictly dependent on the content in the soil [37]. Therefore, it seems right that research assessing the copper content in chokeberry products could be extended to include juices and fibers available on foreign markets and other chokeberry products. This would allow for a comprehensive assessment of the copper content.

Se occurs in the body in the form of selenoproteins. It is a component of some enzymes such as glutathione peroxidase, deiodinase, selenoprotein-P or thioredoxin reductase. It plays an important role in antioxidant defense, thyroid hormone production, DNA synthesis and the body’s reproductive functions. It also participates in the proper functioning of the immune system by stimulating the formation of antibodies and the activity of T lymphocytes (helper and cytotoxic) and NK cells [38,39]. The sources of Se are both plant and animal products—nuts (in particular, Brazil nuts), seeds, cereal products, eggs, meat, milk, some vegetables and fruits [39]. The median of Se in our study was 0.0158 mg/kg. In turn, in the study by Pavlovic et al. (2015), Se was below the detection limit for one of the studied chokeberry juices. In the remaining chokeberry juices in this study, the values ranged from 0.72 to 1.73 mg Se/kg juice [23]. These results were higher than in our study. The Se content in fresh chokeberry fruits ranged from 0.21 to 0.28 mg/kg in the study by Pavlovic et al. (2015) or Se was undetectable, as was the case in the study by Cindric et al. (2017) [23,24]. Chokeberry tea in Pavlovic et al.’s (2015) study also contained a higher Se content, 0.26–0.56 mg/kg of infusion, respectively [23]. The Se content in chokeberry fibers was 61.031 µg/kg. The Se content in fibers was higher than in the juices we examined, but lower than in other products mentioned before. The median of FRAP in chokeberry juices in our study was 71.98 mmol/kg. In the study by Tolic et al. (2015), the FRAP antioxidant potential of the studied chokeberry juices had both lower, similar and higher values—the mean content was 59.05 mmol/kg [40]. The value was almost twice as low as in the study by Błaszczak et al. (2017) (134.61 mmol/kg) [41]. In the study by Soural et al. (2019), the antioxidant potential of FRAP is expressed in Trolox units, therefore, it is not possible to compare it with our juices. However, chokeberry juice was in second place among the eight studied juices [42]. The median of FRAP in our chokeberry fibers was 5.11 mmol/kg and it has a lower antioxidant potential than that found in the studied chokeberry juices in our research and in other chokeberry products in Tolic et al.’s study (2015) [40]. It seems that the fiber production process may have promoted the loss of antioxidants.

The median of TPC in all studied chokeberry juices was 3829.00 mg GAE/kg (3285.00–4633.00 mg/kg). Other authors obtained mostly higher concentrations of polyphenols in chokeberry juices, ranging from 3002 in the study by Tolic et al. (2015) to 11,266 mg GAE/kg in the study by Nowak et al. (2022). The authors used the same method as us to assess the polyphenol content [6,40]. The differences may result from various factors such as the origin of the juice, the ripeness of the fruit at harvest, the methods used during juice production or the method of storage [43,44]. Unfortunately, the mentioned studies did not provide information regarding whether these were conventional or organic juices, or whether juices were made from concentrate or not [6,40]. Comparing the concentration of TPC in chokeberry juices to other chokeberry products, chokeberry nectar in the study by Denev et al. (2018) contained both lower and similar polyphenol content compared to the juices in our study (2475.2–3782.1 mg GAE/kg) [45]. Chokeberry syrup had a lower TPC value in the study by Kapci et al. (2013), 2600 mg GAE/kg, respectively. Fresh chokeberry berries had a high concentration of polyphenols: 19,500–23,400 mg GAE/kg in the Ochmian et al. (2012) study and 13 300 mg GAE/kg in the Kapci et al. study (2013) [46,47]. They compared different chokeberry products and the highest TPC value was observed in chokeberry pomace, then dried chokeberry fruits, chokeberry concentrate, fresh chokeberry fruits, chokeberry jam, chokeberry compote and chokeberry juice, with the lowest TPC value belonged to chokeberry syrup. The differences result from the form of the product, but we noticed that chokeberry juice did not have the highest concentration of polyphenols in this research. The production method and origin of the product may have had an influence. The authors did not take into account the method of fruit cultivation (conventional or ecological) and the juice production method (NFC or FC) [46]. The following juices also had higher TPC compared to our chokeberry juices: black elder juice [6], Japanese quince [48], wild rose [6,48], sea buckthorn, acerola and goji berry [48]. Blackcurrant juices had both lower and higher value of TPC [39,49]. In the study by Nowak et al. (2022), chokeberry juice, among seven tested juices known for their health-promoting properties, had one of the highest polyphenol contents (after wild rose juice) [6]. In turn, strawberry, apple, orange, grapefruit, grape, pineapple, carrot and tomato juices had significantly lower polyphenols content [50,51,52]. TPC value in all chokeberry fibers was 753.50 mg GAE/kg and it is definitely a lower value than in chokeberry juices and other mentioned chokeberry products [6,46,47].

The median of total flavonoids in all studied chokeberry juices was 572.10 mg QE/kg, and in the fibers 55.25 mg QE/kg. The content of total flavonoids in the studies of other authors was expressed in mg of catechins, which makes an accurate comparison impossible. However, we can notice some trends. In the studies by Nowak et al. (2016, 2022), authors compared different types of juices. It was observed that chokeberry juice had a lower total flavonoids content than wild rose juice [6], and higher than sea buckthorn, Japanese quince, elderberry and cranberry juices [6,53]. In the study by Kapci et al. (2013), it was observed that the highest total flavonoids content among all studied chokeberry products belonged to dried chokeberry fruits and this value was significantly higher than in fresh chokeberry fruits. Chokeberry juice had a lower concentration of total flavonoids than dried and fresh fruits, chokeberry jam and chokeberry concentrate, but higher than chokeberry syrup [46]. Horszwald et al. (2013) assessed various techniques for drying chokeberry juice to obtain chokeberry powder. The values they obtained were 34.5–52.2 mg QE/kg chokeberry powder. This value is lower than the median value in the chokeberry juices we studied, but similar to the content of total flavonoids in chokeberry fibers [54]. Both products come in powder form and their production process is probably similar, which is related to the flavonoid content.

Comparing the median total anthocyanins content to chokeberry juices in the studies of other researchers, our chokeberry juices had both lower and higher content. The median in our juices was 197.80 mg Cy-3-O-GL/kg (156.70–297.20 mg/kg). A lower total anthocyanins content was obtained by Konic Ristic et al. (2013), respectively, 150 mg Cy-3-O-GL/kg of commercial chokeberry juice. However, the same authors obtained a much higher value for fresh chokeberry juice, respectively, 2180 mg Cy-3-O-GL/kg of juice. Our study did not include fresh juices—all of them were pasteurized—but the value obtained by Konic Ristic et al. (2013) for commercial juice was slightly lower than for the commercial juices in our study [55]. Tolic et al. (2015) obtained different values, respectively, 150–1228 mg Cy-3-O-GL/kg of chokeberry juice [40]. A higher concentration of total anthocyanins was noted by Kapci et al. (2013)—400–700 mg and by Nowak et al. (2022)—1209 mg Cy-3-O-GL/kg of chokeberry juice [6,46]. Chokeberry juice in the study by Nowak et al. (2022) had a lower content than elderberry juice (1966.8 mg Cy-3-O-GL/L), but higher than wild rose, sea buckthorn, Japanese quince, noni and cranberry juices [6]. As we mentioned earlier, the differences may result from the method of growing the fruit (organic or conventional) used for production (the authors did not provide this information), the ripeness of the fruit at the time of harvest or the method of juice production. The tested fibers also had a lower total anthocyanins value than juices (22.385 mg Cy-3-O-GL/kg, respectively) which may confirm that these antioxidant compounds may be sensitive to the production process used.

Vitamin C is very susceptible to temperature and oxidation [56]. Therefore, its losses may be high depending on the degree of processing of a given product. The median content of the sum of ascorbic and dehydroascorbic acid was 132.66 mg/kg in chokeberry juices and 44.29 mg/kg in chokeberry fibers. The lower value in fiber can confirm the sensitivity of this vitamin to various technological processes. In the study by Catana et al. (2017) and Soural et al. (2019), the authors obtained a much higher vitamin C content for chokeberry juices than in our study: 398.00 [42] and 987.5 mg/kg [57]. Soural et al. (2019) also observed that chokeberry juice had a higher vitamin C content than pomegranate, black elder and aloe vera juices, but significantly lower than acerola, seaberry, cranberry and blackcurrant juices [42]. Apple, grape and orange juices obtained both higher and lower vitamin C values, respectively, 24–32 mg/kg for apple juice [50], 380–454 mg/L for grape juice [58] and 346–612 mg/kg for orange juice [50,58]. Catana et al. (2017) compared various chokeberry products (frozen and dried fruits, jam, compote and fresh chokeberry juice) in terms of vitamin C content and obtained from 72.5 to 987.5 mg/kg of product, with chokeberry juice having the highest content [57].

We noticed that the mineral content of the studied chokeberry juices was lower than in the studied chokeberry fibers, except for Cu. While the opposite relationship was found in the case of antioxidant potential, polyphenols, total flavonoids and total anthocyanins. Juices had a higher content of these antioxidant components. As we mentioned, this may be due to the sensitivity of these antioxidant substances to production processes, as well as the composition of the fiber itself. Differences in the content of individual tested ingredients in all chokeberry products from other authors, including juices, may result from many factors (cultivation method, production method, product storage method and research method used). Unfortunately, we could not compare the influence of cultivation methods (organic or conventional) and the origin of the juice (NFC or FC) because other authors did not take into account this information. However, it can be noted that chokeberry products have a high nutritional value and can be recommended in the diet.

## 4. Conclusions

Chokeberry products such as chokeberry juice and chokeberry fibers are an excellent source of antioxidants, especially polyphenols. However, they also provide other important ingredients such as Mg, trace elements (Cu, Mn, Se and Zn) and vitamin C. These ingredients are extremely valuable in our diet and together they create a rich composition in chokeberry products. Thanks to their valuable properties, they can counteract oxidative stress and, thus, be recommended in the prevention and treatment of many diseases.

## Figures and Tables

**Table 1:** Mineral content in studied chokeberry juices.

Type of Chokeberry Juices		Mg (mg/kg)	Microelements				
			Cu (mg/kg)	Fe (mg/kg)	Mn (mg/kg)	Se (µg/kg)	Zn (mg/kg)
Conventional (*n* = 10)	Med.	151.07	78.38	1.23	4.23	17.02	0.56
	Q1–Q3	136.64–163.66	26.59–89.30	0.84–2.00	3.45–5.15	11.64–21.64	0.49–0.69
	Av. ± SD	150.80 ± 26.85	72.81 ± 47.22	1.40 ± 0.78	4.03 ± 1.54	18.60 ± 8.65	0.57 ± 0.12
	Min-Max	108.65–193.12	16.63–169.41	0.31–2.82	1.40–5.82	8.75–38.73	0.33–0.70
Organic (*n* = 15)	Med.	152.84	61.01	0.92	3.63	15.62	0.60
	Q1–Q3	135.98–199.78	30.74–105.92	0.57–1.19	2.44–4.28	11.48–26.86	0.51–0.77
	Av. ± SD	160.63 ± 37.43	88.09 ± 83.22	1.19 ± 1.20	3.51 ± 1.42	17.91 ± 10.02	0.66 ± 0.21
	Min–Max	105.18–227.21	14.95–317.70	0.31–5.24	1.36–6.49	<dl-40.84	0.41–1.15
NFC (*n* = 20)	Med.	148.40	63.32	0.98	3.70	18.66	0.62
	Q1–Q3	134.61–179.43	28.66–101.59	0.57–1.29	2.39–4.94	12.66–25.68	0.52–0.73
	Av. ± SD	155.79 ± 36.77	84.86 ± 76.91	1.25 ± 1.14	3.66 ± 1.63	19.23 ± 10.04	0.64 ± 0.19
	Min–Max	105.18–227.21	14.95–317.70	0.31–5.24	1.36–6.49	<dl-40.84	0.33–1.15
FC (*n* = 5)	Med.	158.12	80.47	1.39	3.89	15.62	0.49
	Q1–Q3	155.43–161.68	51.22–89.88	0.92–1.70	3.81–4.21	11.64–15.78	0.49–0.69
	Av. ± SD	160.33 ± 15.04	70.47 ± 36.01	1.37 ± 0.50	3.92 ± 0.33	14.00 ± 3.78	0.54 ± 0.09
	Min–Max	142.50–183.92	19.07–111.07	0.83–2.00	3.45–4.25	8.75–18.26	0.48–0.69
Total (*n* = 25)	Med.	152.84	65.64	1.04	3.81	15.78	0.59
	Q1–Q3	136.64–165.74	30.74–97.25	0.71–1.39	2.56–4.52	11.64–21.47	0.49–0.70
	Av. ± SD	156.70 ± 33.34	81.98 ± 70.24	1.28 ± 1.04	3.72 ± 1.46	18.19 ± 9.31	0.62 ± 0.18
	Min–Max	105.18–227.21	14.95–317.70	0.31–5.24	1.36–6.49	<dl-40.84	0.33–1.15

Av.—average, FC—from concentrated, Max—maximum value, Med—median, Min—minimum value, NFC—not from concentrated, Q1—lower quartile, Q3—upper quartile, SD—standard deviation.

**Table 2:** Antioxidants properties of studied chokeberry juices.

Type of Chokeberry Juices		Total Anthocyanins (mg Cy-3-GL/kg)	Total Flavonoids (mg QE/kg)	FRAP (mmol/kg)	TPC (mg GAE/kg)	Vitamin C (mg/kg)
Conventional (*n* = 10)	Med.	193.95	571.15	65.25 *	3718.00	120.17
	Q1–Q3	179.40–213.20	537.90–629.60	56.81–68.25	3516.00–3956.00	98.06–167.00
	Av. ± SD	196.01 ± 26.84	585.65 ± 68.18	64.37 ± 13.38	3752.20 ± 366.25	128.49 ± 40.61
	Min-Max	156.70–248.70	507.90–726.90	41.79–85.66	3285.00–4633.00	72.51–187.32
Organic (*n* = 15)	Med.	214.40	646.30	75.56 *	3918.00	136.78
	Q1–Q3	183.40–239.20	540.00–717.70	71.98–84.30	3783.00–4154.00	106.30–174.69
	Av. ± SD	214.08 ± 37.81	640.91 ± 123.54	78.47 ± 10.84	3969.13 ± 313.39	143.96 ± 54.04
	Min–Max	171.20–297.20	511.50–947.90	56.14–97.41	3375.00–4566.00	66.74–276.32
NFC (*n* = 20)	Med.	213.80 *	637.95 *	75.12 *	3937.00 *	134.72
	Q1–Q3	186.10–236.15	552.40–693.45	71.48–84.18	3782.50–4123.00	101.76–175.51
	Av. ± SD	213.40 ± 35.50	638.69 ± 110.58	77.79 ± 9.79	3986.10 ± 305.31	140.73 ± 51.81
	Min–Max	156.70–297.20	511.50–947.90	64.25–97.41	3516.00–4633.00	66.74–276.32
FC (*n* = 5)	Med.	181.20 *	540.00 *	56.14 *	3375.00 *	127.45
	Q1–Q3	179.40–183.40	537.90–540.20	53.06–56.81	3332.00–3607.00	112.89–149.97
	Av. ± SD	180.68 ± 7.54	539.28 ± 22.12	52.98 ± 6.46	3467.40 ± 195.48	125.96 ± 36.37
	Min–Max	169.30–190.10	507.90–570.40	41.79–57.12	3285.00–3738.00	72.51–167.00
Total (*n* = 25)	Med.	197.80	572.10	71.98	3829.00	132.66
	Q1–Q3	181.20–227.90	540.00–661.50	66.24–82.35	3698.00–3975.00	105.47–167.55
	Av. ± SD	206.85 ± 34.43	618.80 ± 106.82	72.83 ± 13.62	3882.36 ± 353.56	137.77 ± 48.80
	Min–Max	156.70–297.20	507.90–947.90	41.79–97.41	3285.00–4633.00	66.74–276.32

Av.—average, Cy-3-GL—cyanidin-3-glucoside, FC—from concentrated, FRAP—ferric reducing antioxidant power assay, GAE—gallic acid, Max—maximum value, Med—median, Min—minimum value, NFC—not from concentrated, Q1—lower quartile, Q3—upper quartile, QE—quercetin, SD—standard deviation and *—statistically significant difference (*p* < 0.05).

**Table 3:** Content of Mg and microelements in studied chokeberry fibers.

Type of Chokeberry Fiber		Mg (mg/kg)	Microelements				
			Cu (mg/kg)	Fe (mg/kg)	Mn (mg/kg)	Se (µg/kg)	Zn (mg/kg)
Conventional (*n* = 3)	Med.	1038.42	7.08	280.10	37.64	57.36	0.56
	Q1–Q3	826.83–1250.00	5.97–8.19	112.23–447.96	34.62–40.67	50.01–495.02	0.49–0.69
	Min–Max	826.83–1250.00	5.97–8.19	112.23–447.96	34.62–40.66	50.01–495.02	0.33–0.70
	Av. ± SD	1038.42 ± 211.59	7.08 ± 1.11	280.10 ± 167.87	37.638 ± 3.02	200,794 ± 254.83	0.57 ± 0.12
Organic (*n* = 3)	Med.	990.88	5.94	287.58	31.68	64.70	0.60
	Q1–Q3	971.70–1431.00	3.68–6.38	60.31–359.11	31.68–38.61	32.76–212.91	0.51–0.77
	Min-Max	971.70–1431.00	3.68–6.38	60.31–359.11	31.68–38.61	32.76–212.91	0.41–1.15
	Av. ± SD	1131.19 ± 259.82	5.33 ± 1.44	235.67 ± 156.01	33.99 ± 4.00	103.46 ± 96.13	0.66 ± 0.21
Total (*n* = 6)	Med.	1014.65	6.17	283.84	36.13	61.03	17.13
	Q1–Q3	971.70–1250.00	3.68–8.19	112.23–359.11	31.68–38.61	50.01–212.91	12.08–24.64
	Min–Max	826.83–1431.00	3.68–8.19	60.31–447.96	31.68–40.66	32.75–495.02	9.37–84.62
	Av. ± SD	1084.80 ± 217.92	6.205 ± 1.50	257.88 ± 146.97	35.81 ± 3.75	152.13 ± 180.32	0.62 ± 0.18

Av.—average, Max—maximum value, Med—median, Min—minimum value, Q1—lower quartile, Q3—upper quartile, SD—standard deviation.

**Table 4:** Antioxidants properties of chokeberry fibers.

Type of Chokeberry Fiber		Total Anthocyanins (mg Cy-3-GL/kg)	Total Flavonoids (mg QE/kg)	FRAP (mmol/kg)	TPC (mg GAE/kg)	Vitamin C (mg/kg)
Conventional (*n* = 3)	Med.	20.12	46.60	4.47	715.00	39.14
	Q1–Q3	15.59–24.65	40.60–52.60	4.35–4.59	688.00–742.00	37.90–40.38
	Min–Max	15.59–24.65	40.60–52.60	4.35–4.59	688.00–742.00	37.90–40.38
	Av. ± SD	20.12 ± 4.53	46.60 ± 6.00	4.47 ± 0.12	715.00 ± 27.00	39.14 ± 1.24
Organic (*n* = 3)	Med.	26.11	58.90	6.34	774.00	55.21
	Q1–Q3	17.51–28.36	57.90–63.70	5.62–6.51	765.00–785.00	48.20–56.03
	Min–Max	17.51–28.36	57.90–63.70	5.62–6.51	765.00–785.00	48.20–56.03
	Av. ± SD	24.00 ± 5.73	60.17 ± 3.10	6.16 ± 0.47	774.67 ± 10.02	53.15 ± 4.30
Total (*n* = 6)	Med.	22.39	55.25	5.11	753.50	44.29
	Q1–Q3	17.51–26.11	46.60–58.90	4.47–6.34	715.00–774.00	39.14–55.21
	Min–Max	15.59–28.36	40.60–53.70	4.35–6.51	688.00–785.00	37.90–56.03
	Av. ± SD	22.06 ± 5.08	53.38 ± 8.57	5.31 ± 0.97	477.83 ± 37.41	46.14 ± 8.18

Av.—average, Cy-3-GL—cyanidin-3-glucoside, FRAP—ferric reducing antioxidant power assay, GAE—gallic acid, Max—maximum value, Med—median, Min—minimum value, Q1—lower quartile, Q3—upper quartile, QE—quercetin, SD—standard deviation.

**Table 5:** Percentage of reference intake value of studied elements.

Percent of Reference Intake Value (%, Minimum—Maximum)							
Type of Product	Cu	Fe	Mn	Mg	Se	Zn	Vitamin C
Conventional Juices	166.3–1694.1	0.2–2.0	7.0–29.1	2.9–5.1	1.6–7.0	0.3–0.7	9.1–23.4
Organic Juices	149.5–3177.0	0.2–3.7	6.8–32.4	2.8–6.1	0–7.4	0.4–1.2	8.3–34.5
NFC Juices	149.5–3177.0	0.2–3.7	6.8–32.4	2.8–6.1	0–7.4	0.3–1.2	8.3–34.5
FC Juices	190.7–1116.9	0.6–1.4	17.2–21.3	3.8–4.9	1.6–3.3	0.5–0.7	9.1–20.9
Juices (Total)	149.5–3177.0	0.2–3.7	6.8–32.4	2.8–6.1	0.0–7.4	0.3–1.2	8.3–34.5
Conventional Fibers	6.0–8.2	8.0–32.0	17.3–20.3	2.2–3.3	0.9–9.0	0.9–2.5	0.5
Organic Fibers	3.7–6.4	4.3–25.7	15.8–19.3	2.6–3.8	0.6–3.9	1.2–8.5	0.6–0.7
Fibers (Total)	3.7–8.2	4.3–32.0	15.8–20.3	2.2–3.8	0.6–9.0	0.9–8.5	0.5–0.7

## Data Availability

The data used to support the findings of this study can be made available by the corresponding author upon request.

## Supplementary Materials

The following supporting information can be downloaded at: https://www.mdpi.com/article/10.3390/foods12214029/s1, Table S1: Content of studied antioxidants parameters in other studies.
