# Supplementary material for: Antioxidant Properties of Chokeberry Products—Assessment of the Composition of Juices and Fibers
Source: Foods. 2023 Nov 5;12(21):4029. doi: 10.3390/foods12214029 (PMC10649922; doi:10.3390/foods12214029)
Supplement: Supplementary file 1 [file foods-12-04029-s001.zip › foods-2680829-supplementary.pdf]

Article

Antioxidant Properties of Chokeberry Products - Assessment of the Composition of Juices and Fibers

Ewa Olechno [1], Anna Puścion-Jakubik [2,*], Jolanta Soroczyńska [2], Katarzyna Socha [2], Monika Cyuńczyk [1], Małgorzata Elżbieta Zujko [1,*]

[1] Department of Food Biotechnology, Faculty of Health Science, Medical University of Białystok, Szpitalna 37 Street, 15-295 Białystok, Poland; ewa.olechno@sd.umb.edu.pl (E.O.); monika.cyunczyk@umb.edu.pl (M.C.); malgorzata.zujko@umb.edu.pl (M.E.Z.)

[2] Department of Bromatology, Faculty of Pharmacy with the Division of Laboratory Medicine, Medical University of Białystok, Mickiewicza 2D Street, 15-222 Białystok, Poland; anna.puscion-jakubik@umb.edu.pl (A.P.J.); jolanta.soroczynska@umb.edu.pl (J.S.); katarzyna.socha@umb.edu.pl (K.S.)

* Correspondence: anna.puscion-jakubik@umb.edu.pl, malgorzata.zujko@umb.edu.pl

Table S1. Content of Studied Antioxidants Parameters in Other Studies.

Studied Component	Type Of Product	Content of Studied Antioxidant Parameters
Total Anthocyjanins in Chokeberry Products (mg Cy-3-GL/kg of Product)	Chokeberry Juice	1209.7 [6], 150-1228 [40], 400-700 [46], 150 (Commercial Chokeberry Juice) [55], 2180 (Fresh Chokeberry Juice) [55]
	Chokeberry Fruits	6936 [43], 6784-7415 [44], 13087.5-14552.5 [57], 4500 [46]
	Chokeberry Dried Fruits	1954-2466 [40] 1400-3100 [46]
	Chokeberyy Concentrate	3600 [46]
	Chokeberry Pomace	10000 [46]
	Chokeberry Jam	200-400 [46]
	Chokeberry Compote	200 [46]
	Chokeberry Syrup	100 [46]
	Chokeberry Powder	1165-1641 [40]
	Chokeberry Fruit Tea	282-675 [40]
Total Anthocyjanins in Other Products (mg Cy-3-GL/kg of Product)	Acerola Juice	55.7 [48]
	Cranberry Juice	200.4 [48]
	Elderberry Juice	1966.8 [6]
	Gojiiberry Juice	44.5 [48]
	Japanese Quince Juice	77.9 [48]
	Sea Buckthorn Juice	22.3 [48]
	Wild Rose Juice	0.3 [6], 27.8 [48]
	Bilberry Fruits	6555 [43]
	Black Elder Fruits	7183 [43]
	Raspberry Fruits	598 [43]
Total Flavonoids in Chokeberry Products (mg CE or QE/kg of Product)	Chokeberrry Juice	2700-2900[a] [46], 5069[a] [6]
	Chokeberrry Fruits	5300[a] [46]
	Chokeberrry Powder	34.5-52.2[b] [54]
	Chokeberrry Dried Berries	12500-19900[a] [46]
	Chokebery Concentrate	6100[a] [46]
	Chokeberry Pomace	9300[a] [46]
	Chokeberry Jam	2900-6400[a] [46]
	Chokeberry Compote	3300[a] [46]

Total Flavonoids in Other Products (mg QE/kg of Product)	Acerola Juice	1574[a] [48]
	Cranberry Juice	24[a] [6], 527[a] [48]
	Elderberry Juice	804[a] [6]
	Gojiberry Juice	139[a] [48]
	Japanese Quince Juice	3214[a] [6], 2641[a] [48]
	Sea Buckthron Juice	3833[a] [6], 251[a] [48]
	Wild Rose Juice	5607[a] [6], 5864[a] [48]
FRAP in Chokeberry Products (mmol/kg)	Chokeberry Juice	7.79-10.41[d] [13], 38.71-79.86[c] [40], 134.61[c] [41], 44.9[e] [42]
	Dried Chokeberry Fruits	183.52-191.31[c] [40]
	Chokeberry Powder	47.38-68.6[c] [40]
	Chokeberry Fruit Tea	111.43-163.33[c] [40]
FRAP in Other Products (mmol/kg)	Acerola Juice	63.2[e] [42]
	Blackcurrant Juice	36.5[e] [42]<br>108[c] [49]
	Black Elder Juice	35.5[e] [42]
	Cranberry Juice	7.5[e] [42]
	Pomegranate Juice	28.7[e] [42]
	Sea Buckthorn Juice	11.6[e] [42]
TPC in Chokeberry Products (mg GAE/kg)	Chokeberry Juice	11,266 [6], 3002-6639 [40], 8297 [42], 6300-6600 [46], 5138-8988.3 [45], 5561-7956 [53], 5860 -5930 [55], 6884.7 [57]
	Chokeberry Nectar	2475.2-3782.1 [45]
	Chokeberry Fresh Berries	19,500-23,400 [47], 13,300 [46]
	Chokeberry Dried Berries	1954-2466 [40], 40152.5 [57] 39,900-50,100 [46]
	Chokebery Concentrate	29,600 [46]
	Chokeberry Pomace	9300 [46]
	Chokeberry Jam	6900-12000 [46]
	Chokeberry Compote	6700 [46]
	Chokeberry Syrup	2600 [46]
	Chokeberry Tea	1494-3436 [40]
	Chokeberry Powder	276.3-326.1 [54], 4233-4434 [40]
	Acerola Juice	13707 [48], 7362 [42]

TPC in Other Products (mg GAE/kg)	Apple Juice	571 [50], 1142.12 [51]
	Black Elder Juice	5881 [42]
	Billberry Juice	3845.04 [51]
	Blackcurrant Juice	1088.8-1392.2 [52], 5402 [42]
	Carrot Juice	338.9 [52]
	Cranberry Juice	2773 [6], 1627 [53], 3504 [48], 1547 [42]
	Gojiiberry Juice	4028 [48]
	Grape Juice	1674.79 [51], 643.4-1564 [52]
	Grapefruit juice	496.8-537.6 [52]
	Japanese Quince	6852 [48]
	Orange Juice	840.59 [51], 385.1-541.6 [52], 195 [50]
	Pineapple juice	369.6-1018.6 [52]
	Pomegranate Juice	3558 [53], 3717 [42]
	Sea Buckthorn Juice	4784 [48], 2408 [42]
	Sour CherryJuice	2447.25 [51]
	Strawberry Juice	1406.12 [51]
	Tomato Juice	213.6-358.1 [52], 184 [50]
	Wild Rose Juice	16,619 [6], 13188 [48]
	Elderberry Juice	6826 [6], 3239 [53]
Vitamin C in Chokeberry Products (mg AA/kg)	Chokeberry Juice	398 [42], 987.5 [53]
	Chokeberry Fruits	130 [42], 318.5 [53]
	Chokeberry Dried Berries	151.1 [53]
	Chokeberry Jam	79.6 [53]
Vitamin C in Other Products (mg AA/kg)	Acerola Juice	9283 [48], 8528 [42]
	Apple Juice	372 [50]
	Apple, Puree	149-435 [50]
	Bilberry Fruits	183 [43]
	Blackcurrant Juice	801 [42]
	Black Elder Juice	295 [42]
	Black Elder Fruits	171 [43]
	Cranberry Juice	250 [48], 1145 [42]
	Gojiiberry Juice	4237 [48]
	Grape Juice	380.16-454.57 [58]

	Japanese Quince Juice	1978 [48]
	Orange Juice	346 [50], 550.87-612.15 [58]
	Pomegranate Juice	5 [42]
	Raspberry Fruits	200 [43]
	Sea Buckthorn Juice	3255 [48], 1175 [42]
	Tomato Juice	32 [50]
	Wild Rose Juice	4548 [48]

AA - ascorbic acid, Cy-3-GL - cyanidin-3-glucoside, FRAP - Ferric Reducing Antioxidant Power Assay, GAE - gallic acid, QE- quercetin, [a] - mg catechin/kg of product , [b] – mg quercetin/kg of product , [c] – FRAP value in mmol/kg of product, [d] – FRAP value in mg of Trollox equivalents/mL of product, [e] - FRAP value in mM of Trollox equivalents
